# Patients with obesity have more inflamed joints and higher CRP levels during the disease course in ACPA-positive RA but not in ACPA-negative RA

**DOI:** 10.1186/s13075-023-03248-8

**Published:** 2024-02-07

**Authors:** N. K. den Hollander, A. M. P Boeren, A. H. M. van der Helm-van Mil, H. W. van Steenbergen

**Affiliations:** 1 Department of Rheumatology, Leiden University Medical Center, P.O. Box 9600, 2300 RC Leiden, The Netherlands; 2 Department of Rheumatology, Erasmus Medical Center, Rotterdam, The Netherlands

## Abstract

**Background:**

Obese RA patients have higher disease activity scores (DAS). Previous research showed that obese RA patients have higher tender joint count (TJC) and VAS general health. However, it remains unclear whether DAS components measuring local and systemic inflammation (swollen joint count (SJC), CRP) are increased and if this is present in the total RA population or confined to an ACPA subgroup. As ACPA is suggested to enhance inflammatory responses, we hypothesized that the association of obesity with SJC and CRP is present especially in ACPA-positive RA. We therefore studied associations of obesity with courses of DAS components in ACPA subgroups.

**Methods:**

We studied 649 RA patients (291 ACPA-positive), included in the Leiden Early Arthritis Clinic. Five-year courses of DAS44 and DAS44 components (SJC—44, TJC—53, CRP, VAS (0–100)) were compared between RA patients with normal weight (BMI 18.5–24.9), overweight (25.0–29.9), and obesity (≥ 30.0), stratified for ACPA. Linear/Poisson mixed models with a knot at 4 months were used.

**Results:**

Obese RA patients had + 0.32 higher DAS compared to normal weight during the 5-year follow-up. In ACPA-positive RA, obese patients had + 0.43 (95% CI: 0.22, 0.64) higher DAS, whereas in ACPA-negative RA, this difference was smaller and not statistically significant: + 0.19 (95% CI: − 0.01, 0.38). In ACPA-positive RA, all DAS components were significantly higher in obese patients compared to normal weight: SJC + 60% (IRR1.60; 95% CI: 1.18, 2.16), CRP + 3.7 mg/L (95% CI:0.95, 6.53), TJC + 55% (IRR1.55; 95% CI:1.15, 2.10), and VAS + 9 (95% CI: 4.0, 14.2). ACPA-negative obese RA patients tended to have higher TJC (IRR1.22; 95% CI: 0.96, 1.55) and VAS (β4.3; 95% CI: − 0.4, 9.0), while SJC (IRR1.07; 95% CI:0.85, 1.33) and CRP (β0.24; 95% CI: − 1.29, 3.32) were unaffected.

**Conclusion:**

The association of obesity with a worse DAS course is mainly present in ACPA-positive RA; especially SJC and CRP levels remain higher in ACPA-positive RA patients with obesity but not ACPA-negative RA patients. This is the first demonstration that obesity influences the disease course of ACPA-positive and ACPA-negative RA differently.

**Supplementary Information:**

The online version contains supplementary material available at 10.1186/s13075-023-03248-8.

## Introduction

Rheumatoid arthritis (RA) patients with obesity have higher disease activity scores (DAS), have lower odds of achieving disease remission, and are less likely to maintain sustained remission compared to RA patients with normal weight [1–4]. Multiple studies showed that the DAS components tender joint count (TJC) and VAS general health are higher in patients suffering from obesity, reflecting more pain and worse general health in patients with obesity [1, 4]. However, results of previous studies are conflicting about whether obesity results in higher swollen joint count (SJC) and CRP levels, which could represent local and systemic inflammation. As adipose tissue is assumed to have immunomodulating and proinflammatory properties, obesity could potentially increase local and systemic inflammation as measured by SJC and CRP [5].

In addition to obesity, in vitro data suggested that auto-antibodies such as ACPA could also enhance inflammatory responses by increasing TNF-α production and complement system activation [6, 7]. This could contribute to the more severe disease course in ACPA-positive RA patients compared to ACPA-negative RA patients [8, 9]. However, it is unclear whether obesity affects disease activity, local and systemic inflammation similarly in ACPA-positive and ACPA-negative RA.

To increase our understanding of the effect of obesity on disease activity in relation to ACPA, we studied if patients with obesity (1) have a higher DAS during disease course and if this is similar for ACPA-positive and ACPA-negative RA and (2) if this is true for the course of all individual DAS components, with a main focus on the SJC and CRP as these reflect local and systemic inflammation.

## Patients and methods

### Patients

The Leiden Early Arthritis Clinic (EAC) is a population-based inception cohort consecutively including all newly presenting patients with recent onset arthritis of at least one joint and a symptom duration of less than 2 years. This cohort is described in detail elsewhere [10]. The study was approved by the local medical ethics committee of the Leiden University Medical Centre (“Commissie Medische Ethiek,” B19.008). All patients provided written informed consent.

For the current study, only patients with RA who were included from June 2011 onwards were assessed (Supplementary Fig. 1). This time period was chosen to assure that similar treatments and treatment strategies were used in all assessed patients. RA was defined as fulfillment of 2010 or 1987 RA classification criteria [11, 12]. Both 2010 and 1987 RA classification criteria were considered in the definition of RA, as autoantibody-positive RA patients are identified earlier using the 2010 RA classification criteria but autoantibody-negative RA can be classified earlier using the 1987 classification criteria since autoantibody-negative patients need > 10 involved joints to fulfill the 2010 RA classification criteria [13].

At the first visit, patients and rheumatologists completed questionnaires, physical examination was performed (including measuring weight, height, swollen and tender joint counts), and blood samples were taken for routine laboratory procedures (including ACPA (EliA-CPP (anti-CCP2), Phadia, Nieuwegein, the Netherlands, elevated if ≥ 10 U/ml), IgM-RF (in-house ELISA, elevated if ≥ 5.0 IU/ml), and CRP (mg/l)). Calculated BMI (kg/m^2^) was categorized according to WHO-definitions in normal weight (BMI 18.5–24.9), overweight (BMI 25.0–29.9), and obese (BMI > 29.9).

Follow-up visits were performed at 4, 8, and 12 months during the first year and yearly thereafter, as long as patients were treated at the outpatient clinic. At follow-up visits questionnaires, physical examination and laboratory procedures were repeated. DAS44 was assessed at each visit and consisted of 4 components: SJC consisting of 44 joints (SJC-44), CRP, TJC consisting of 53 joints (TJC-53), and visual analog scale general health (VAS, ranging from 0 to 100 where 0 represents excellent general health, 100 poor general health). With these components, DAS44 was calculated by using the formula (0.53938 × √(TJC44)) + (0.06465 × SJC44) + (0.17 × LN (CRP + 1)) + (0.00722 × VAS general health) + 0.45) [14]. DAS44 was chosen over DAS28, as the DAS44 also included the feet. Follow-up ended in case of release from care due to prolonged sustained DMARD-free remission, withdrawal of informed consent while remaining treated, or death. Withdrawal was rare, as most data was collected at regular visits to the rheumatologist in the outpatient clinic.

### Statistical analyses

The effect of baseline BMI on the courses of DAS and DAS components was studied using mixed models. A spline at 4 months was added, based on both the structured research visits and previous research from our group showing that DAS declined the strongest in the first 4 months [15]. Linear mixed models were used for analyses of the course of DAS44, CRP, and VAS. Poisson mixed models were used for analyses of the course of SJC and TJC, as these are count data. Mixed models are suitable for longitudinal measurements since they adjust for within-patient effects. Individual DAS components were analyzed for ACPA-positive and ACPA-negative patients separately. In all models, either DAS44 or one of the DAS components was the dependent variable (outcome), and categorized baseline BMI was the independent variable with normal weight as reference. The spline for time in months was added for the period 0–4 months and 4–60 months (5 years). Results of linear mixed models were represented as betas on the additive scale; thus, a beta of < 0 indicates lower DAS/CRP/VAS, and a beta of > 0 indicates a higher DAS/CRP/VAS compared to the reference. As Poisson mixed models incorporate log-transformation, results for SJC and TJC were represented as incidence rate ratios (IRR) on the multiplicative scale. An IRR of < 1 indicates relatively lower SJC/TJC, and an IRR of > 1 indicates relatively higher SJC/TJC compared to the reference. All analyses were corrected for age and sex. IBM SPSS Statistics v25 and StataCorp Stata v.16 were used.

### Sensitivity analysis

To assess the robustness of our results, a sensitivity analysis was performed. In this sensitivity analysis, DAS and DAS components were assessed separately for autoantibody-positive and autoantibody-negative patients, where autoantibody-positivity was defined as ACPA-positive and/or RF-positive (rather than ACPA-positivity only).

## Results

### Study population

A total of 649 RA patients were consecutively included from June 2011 until July 2021 and studied, of whom 291 were ACPA-positive (Supplementary Fig. 1). Baseline characteristics are presented in Table 1. Mean age was 59.7 years, 63% were female patients, median BMI was 26.0, and median DAS was 3.1. ACPA-positive patients had a lower median BMI (25.4 versus 26.4) and DAS(2.8 versus 3.3) compared to ACPA-negative patients.

**Table 1:** Baseline characteristics for ACPA-positive and ACPA-negative patients

	Total *N* = 649	ACPA-positive *N* = 291	ACPA-negative *N* = 358
Age at inclusion	59.7 (14.2)	57.2 (14.2)	61.8 (13.9)^*^
Female sex	63%	60%	65%
SJC (44 joints)	5 (2–10)	5 (2–8)	6 (3–11)^*^
TJC (53 joints)	8 (4–13)	6 (3–10)	9 (5–15)^*^
CRP (mg/L)	9.0 (3–24)	8.7 (4–19)	10.0 (3–26)
ACPA-positive and RF-positive	36%	82%	0%^*^
Only ACPA-positive	6%	14%	0%^*^
Only RF-positive	13%	0%	23%^*^
VAS general health (mm)	40 (20–60)	40 (20–60)	50 (30–70)^*^
DAS44 CRP	3.1 (2.4–3.7)	2.8 (2.2–3.4)	3.3 (2.6–3.9)^*^
BMI (kg/m^2^)	26.0 (23.3–29.0)	25.4 (23.1–28.5)	26.4 (23.7–29.5)^*^
BMI categorized			
*Normal BMI 18.5*–*24.9*	40%	44%	36%
*Overweight 25*–*29.9*	40%	38%	42%
*Obese > 29.9*	19%	16%	21%

Data are presented as mean (SD) or median (IQR) for continuous variables and in percentage (%) for categorical variables. *n* = 7 patients suffered from underweight

*Abbreviations*: *ACPA* Anti-citrullinated protein antibodies, *SJC* Swollen joint count, *TJC* Tender joint count, *CRP* C-reactive protein, *RF* Rheumatoid factor, *VAS* Visual analog scale, *DAS* Disease activity score, *BMI* Body mass index

^*^Significant difference between ACPA-positive and ACPA-negative patients (*p*-value < 0.05)

### Obesity and DAS course in RA patients and ACPA subgroups

**Fig. 1:**
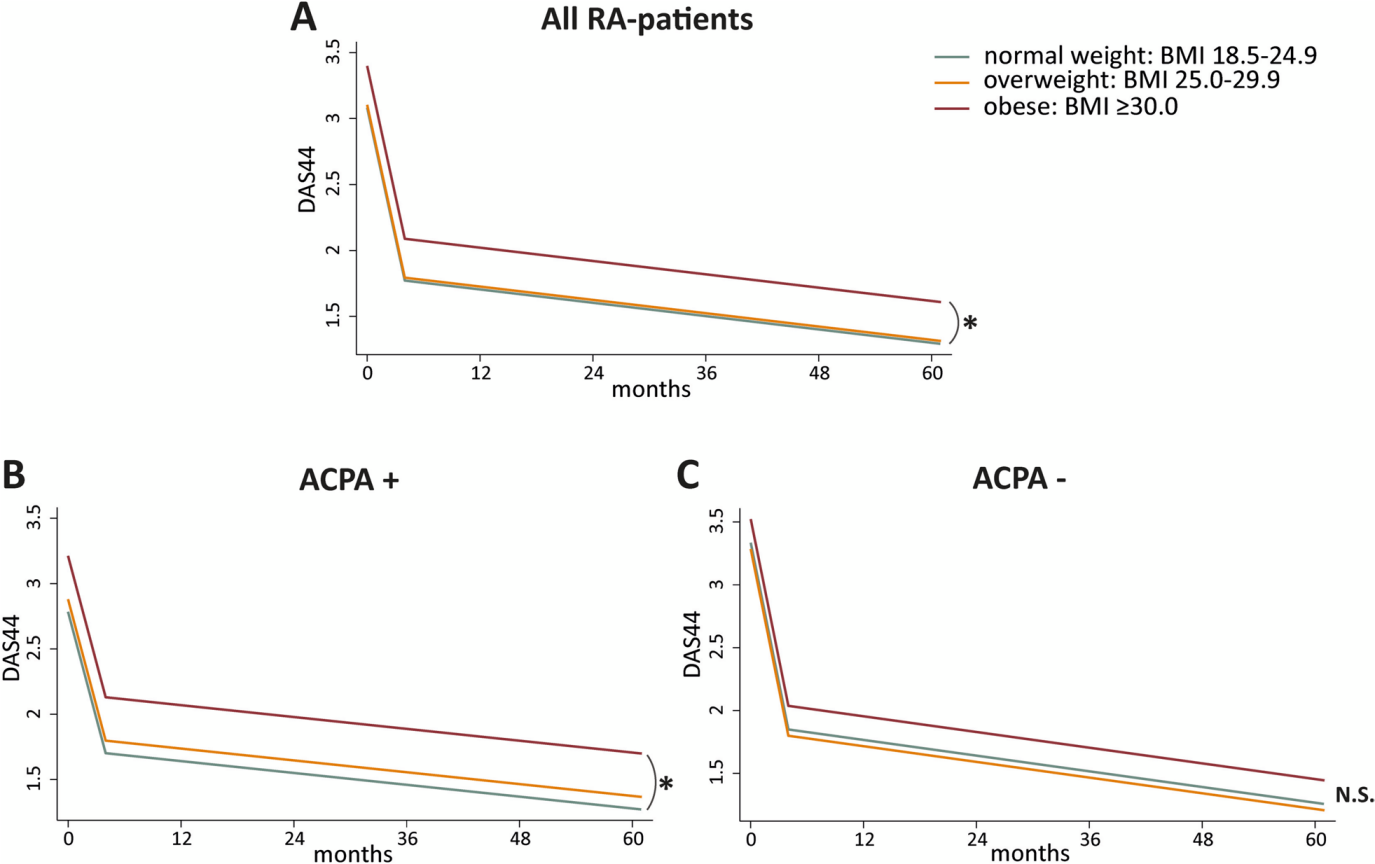
Obesity in RA significantly associates with higher DAS courses, especially in ACPA-positive RA Trajectories of DAS44 per BMI category for all RA patients (**A**), ACPA-positive (**B**), and ACPA-negative RA patients (**C**). All trajectories are shown for mean age of patients per population (overall RA, ACPA-positive and ACPA-negative separately). The number of measurements per time point is presented in Supplementary Fig. 3; the distribution of the patients between the different BMI categories was similar during the 5-year follow-up. **A** RA patients with obesity: + 0.31 points higher DAS (95% CI 0.17, 0.45; *p*-value < 0.001). **B** ACPA-positive patients with obesity: + 0.43 points higher DAS (95% CI 0.23, 0.64; *p*-value 0.002) compared to normal weight. **C** ACPA-negative patients with obesity: trend towards + 0.16 higher DAS compared to normal weight (95% CI − 0.03, 0.36; *p*-value 0.62). Patterns were visualized based on estimated marginal means resulting from the linear mixed models. *p-value < 0.05, compared with normal weight patients. Abbreviations: ACPA, anti-citrullinated protein antibodies; DAS, disease activity score; BMI, body mass index, N. S not significant

Our first aim was to study the association of obesity and the course of DAS during the 5-year follow-up. Although all DAS courses significantly decreased, patients with obesity had a 0.31 (95% CI 0.17, 0.45) higher DAS compared to normal weight patients at diagnosis and during the entire follow-up (Fig. 1A). The DAS course of patients with overweight did not significantly differ from normal weight patients. Thus, similar to previous research, we found that patients with obesity have a significantly higher DAS course than normal weight patients.

As we were especially interested in whether the association between obesity and DAS course differed between ACPA-subgroups, we assessed ACPA-positive and ACPA-negative RA patients separately. In ACPA-positive RA, patients with obesity had a significantly higher DAS during the entire 5-year follow-up compared to normal weight patients (β0.43; 95% CI 0.23, 0.64; Fig. 1B, for raw data see Supplementary Fig. 2). In ACPA-negative RA patients however, the difference between DAS for RA patients with obesity and normal weight RA patients was smaller and not statistically significant (β0.16 units; 95% CI − 0.03, 0.36; Fig. 1C). In both ACPA-positive and ACPA-negative RA, the DAS course for patients with overweight did not significantly differ from normal weight patients. Thus, the association between obesity and higher DAS scores in RA patients appears to be predominantly present in ACPA-positive RA patients.

### Obesity and SJC over time

**Fig. 2:**
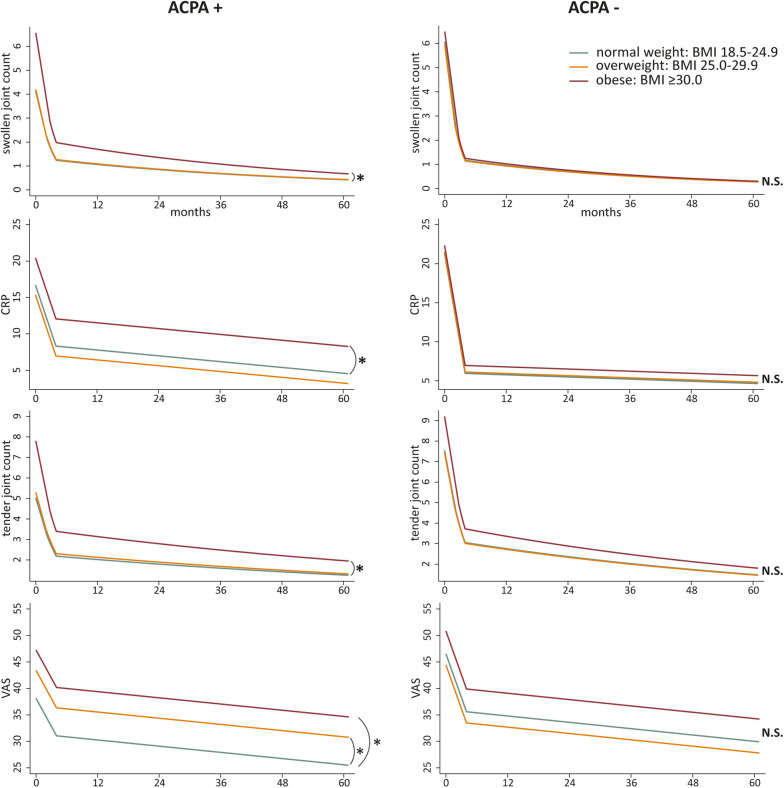
Obesity associates with more swollen joints and higher CRP levels over time in ACPA-positive but not in ACPA-negative RA patients Trajectories of DAS components for ACPA-positive and ACPA-negative RA patients, per BMI-category over 5-year follow-up. All trajectories are shown for mean age per ACPA population. SJC: obese ACPA-positive RA: 59% higher SJC (IRR1.59 95% CI 1.18, 2.15), obese ACPA-negative RA: no significant difference with normal weight (IRR1.05 95% CI 0.85, 1.32). CRP: obese ACPA-positive RA: + 3.7 mg/L higher CRP (95% CI 0.96, 6.52). Obese ACPA-negative RA: no significant difference (β1.02 95% CI − 1.29, 3.33). TJC: obese ACPA-positive RA: 56% higher TJC (IRR1.56 95% CI 1.16, 2.10). Obese ACPA-negative RA: trend towards 18% higher TJC (IRR 1.18 95% CI 0.93, 1.50). VAS: obese ACPA-positive RA: VAS + 9 units (95% CI 4.5, 14.4). Obese ACPA-negative RA: trend towards + 4 units higher VAS (95% CI − 0.8, 8.5). Patterns were visualized based on estimated marginal means resulting from either the linear or Poisson mixed models. *p-value < 0.05, significant difference compared to normal weight. Abbreviations: ACPA, anti-citrullinated protein antibodies; BMI, body mass index; SJC, swollen joint count; CRP, c-reactive protein; TJC, tender joint count; VAS, visual analog scale

Thereafter, we studied all DAS components individually and separately in ACPA-positive and ACPA-negative RA. Firstly, we assessed the association of obesity with the course of SJC. In ACPA-positive RA, patients with obesity had a significantly higher SJC course compared to normal weight patients: IRR 1.59 (95% CI 1.18, 2.15; Fig. 2, for raw data see Supplementary Fig. 2). As IRRs are interpreted on the multiplicative scale, this indicates that patients with obesity had 59% higher SJC at baseline and during the entire follow-up. Patients with overweight had similar SJC courses as normal weight patients.

Contrarily, ACPA-negative RA patients with obesity had a SJC course similar to ACPA-negative RA patients with normal weight: IRR 1.05 (95% CI 0.85, 1.32; Fig. 2). Thus, only ACPA-positive patients with obesity had a higher SJC over time, while ACPA-negative patients with obesity did not.

### Obesity and CRP levels over time

Next, the DAS component CRP, resembling systemic inflammation, was studied. Compared to ACPA-positive normal weight patients, ACPA-positive patients with obesity had 3.7 mg/L higher CRP levels at baseline and during 5-year follow-up (β3.7, 95% CI 0.96, 6.52; Fig. 2; for raw data, see Supplementary Fig. 2). RA patients with overweight did not have higher CRP levels compared to normal weight.

In ACPA-negative RA patients, CRP levels did not significantly differ for patients with overweight or obesity compared with normal weight patients (β0.17, 95% CI − 1.82, 2.15 and β1.02, 95% CI − 1.29, 3.33, respectively; Fig. 2). Thus, while ACPA-positive RA patients with obesity had higher CRP levels over time compared to normal weight RA patients, this did not apply to ACPA-negative RA patients.

### Obesity and TJC over time

Thereafter, the association of BMI-subgroups on the course of TJC was studied for ACPA-positive and ACPA-negative RA separately. In ACPA-positive RA, patients with overweight did not have higher TJC scores compared with normal weight patients. ACPA-positive RA patients with obesity, however, had a 56% higher tender joint count compared to normal weight patients (IRR 1.56; 95% CI 1.16, 2.10; Fig. 2; for raw data, see Supplementary Fig. 2).

In ACPA-negative RA, a statistically non-significant trend towards a 18% higher TJC in patients with obesity was seen (IRR 1.18; 95% CI 0.93, 1.50, Fig. 2). Thus, ACPA-positive RA patients with obesity had a significantly higher TJC during entire follow-up, and ACPA-negative patients with obesity had a tendency towards a higher TJC.

### Obesity and VAS over time

Lastly, VAS general health was studied. In ACPA-positive RA, both patients with overweight and patients with obesity had significantly higher VAS scores compared to normal weight. Patients with overweight had + 6 higher VAS (95% CI 2.5, 10.2), and patients with obesity + 9 (95% CI 4.5, 14.4; Fig. 2; for raw data, see Supplementary Fig. 2).

In ACPA-negative RA, only RA patients with obesity had a trend towards a + 4 higher VAS (95% CI − 0.8, 8.5; Fig. 2). Thus, in ACPA-positive RA, both patients with overweight and obesity had higher VAS scores during the 5-year follow-up, and ACPA-negative patients with obesity showed a statistically insignificant tendency towards higher VAS- scores during the 5-year follow-up.

### Sensitivity analysis in autoantibody subgroups

The sensitivity analysis assessed DAS and DAS components for autoantibody-positive and autoantibody-negative patients separately. In total, 633 patients were studied, of whom 374 were autoantibody-positive. Baseline characteristics are shown in Supplementary Table 1. In autoantibody-positive RA, patients with obesity had a significantly higher course of DAS and all DAS components compared to normal weight patients (Supplementary Fig. 4), similar as seen in ACPA-positive RA. In autoantibody-negative RA, patients with obesity had a significantly higher DAS course compared to normal weight patients; autoantibody-negative patients with obesity had a (not-significant) trend towards a higher TJC course and a significantly higher VAS compared with normal weight patients (both shown in Supplementary Fig. 4). In ACPA-negative patients, a tendency towards a slightly higher TJC course and trend towards a higher VAS in obese patients was also seen. Thus, the results for DAS and DAS components in relation to BMI were similar within the ACPA-positive and ACPA-negative and autoantibody-positive and autoantibody-negative subgroups except the higher VAS in obese patients (which was statistically significant in the ACPA-positive group and a tendency towards being statistically significant in the ACPA-negative group).

## Discussion

In this large longitudinal study, we confirmed that patients with obesity tend to have a higher disease activity during the course of their disease compared to normal weight RA patients, and we identified that this adverse association is only present in ACPA-positive RA. In line with this, the associations between obesity and the course of the individual DAS components differed between ACPA subgroups as well. Within ACPA-positive RA, the courses of all individual DAS components remained more severe in RA patients with obesity compared with normal weight RA patients. This included the “objective measures” reflecting inflammation (SJC,CRP), whereas ACPA-negative RA patients with obesity did not have more inflammation as assessed by SJC and CRP during the disease course compared to normal weight patients. Our study is the first large observational study with repeated DAS measurements during a 5-year follow-up that shows that the effect of obesity on the DAS and its inflammatory components is dissimilar for ACPA-positive and ACPA-negative RA.

Previous studies indicated potential different underlying biological pathways in disease development as well as disease progression between ACPA-positive and ACPA-negative RA [8, 16]. Our study highlights that obesity has a different effect on the disease course of ACPA-positive and ACPA-negative RA patients, which may further support the hypothesis that ACPA-positive and ACPA-negative disease are different subsets of the disease or even different entities (in line with a recently suggested differentiation in type 1 and 2 RA) [8].

SJC and CRP can be considered as proxies of local and systemic inflammation, respectively. Obesity is considered a pro-inflammatory state. Adipose tissue secretes cytokines and adipokines which are known to have pro-inflammatory effects, but it also secretes some mediators that may have anti-inflammatory effects [1]. Based on the data from our study, it may be suggested that the secretion is qualitatively or quantitatively different in ACPA-positive and ACPA-negative RA. Subsequent translational studies are required to fully comprehend the interplay of obesity and inflammatory mediators. Importantly, our results emphasize the relevance of assessing ACPA-positive and ACPA-negative RA separately in such future studies.

In contrast to our study, a previous study suggested that both seropositive and seronegative RA had worse DAS scores in patients with obesity. However, individual points in time were analyzed cross-sectionally during a maximum follow-up of 2 years, and the sample size was lower (*n* = 260) [17]. Our study included more patients (*n* = 649) and serial measurements over 5 years of disease, analyzed with a repeated measurement analysis that can take all measured data into account independent of whether there are some missing values over time, which all increased the power of the current study and enabled well-powered studies within ACPA-positive and ACPA-negative RA separately. In addition, our study is the first large longitudinal study that also included assessment of the course of the individual DAS components per BMI category, which provides a deeper understanding of the differences between ACPA-positive and ACPA-negative RA on this matter [17]. A potential limitation is that our study was not a multi-ethnic study: 95% or our population was Caucasian, which is representative for the local population. However, therefore, we are not able to apply our results regarding high BMI on a non-Caucasian population.

We used BMI to determine patients with obesity. A limitation of this method is that it does not distinguish lean body mass and fat mass. Therefore, BMI might less accurately estimate bodyfat percentage and distribution than alternative techniques (i.e., bioelectrical impedance), which were not available.

It has been suggested that obesity may affect the ability of measuring swollen joints during physical examination and that it may lead to either overestimation or under recognition of SJC [1, 3]. Importantly, SJC was assessed in an identical way in all patients in our study, and thus, a difference between ACPA-positive and ACPA-negative RA would not be affected by difficulties with joint examination, if these would be present. Likewise, patients were treated in line with the recommendations, which do not include different regimens based on BMI. Indeed, in our study, methotrexate was most often used as first DMARD, and the frequency of MTX start was not statistically different for the three BMI categories (Supplementary Table 2). Differences in the association between BMI and DAS/DAS components are therefore unlikely to result from obesity-related treatment differences.

Currently, active disease (persistent inflammation) is the key contributor to RA-related functional disabilities. The finding that obesity is particularly unfavorable with respect to the disease activity in ACPA-positive RA raises the question whether the advice to reduce weight should be particularly stressed among ACPA-positive RA patients with obesity. Additionally, recent studies suggested that obesity might hamper the treatment effect of some biologicals, which could also contribute to differences in disease activity [18, 19]. Current treatment guidelines are similar for ACPA-positive and ACPA-negative RA and do not incorporate BMI in initial treatment decision making. Based on the current findings and knowledge, it can be questioned whether treatment regimens in ACPA-positive RA patients with obesity should be more stringent directly from diagnosis onwards, in order to be able to treat-to-target. More research is needed to study this more thoroughly.

## Conclusion

Obesity keeps increasing worldwide, and since obesity is also a risk factor to develop RA, the population of RA patients with obesity will increase even more [3]. This study with 5-year follow-up showed that obesity had more unfavorable effects on the course of DAS in ACPA-positive RA. Moreover, the presence of obesity at the time of diagnosis is related to persistently higher levels of local and systemic inflammation in ACPA-positive RA but not in ACPA-negative RA. This study is the first to show that the impact of obesity is different for ACPA-positive and ACPA-negative RA.

### Supplementary Information

**Additional file 1: Supplementary Figure 1.** Overview of patient selection. **Supplementary Figure 2.** Raw data of mean DAS44 and DAS-components per ACPA-subgroup per time point and per BMI-category. **Supplementary Figure 3.** The distribution of the patients between the different BMI-categories for ACPA-positive (A) and ACPA-negative (B) patients was similar during the 5-year follow-up. **Supplementary Figure 4.** Obesity associates with higher DAS and DAS-components in autoantibody positive patients and only with higher DAS44 and VAS in autoantibody negative patients. **Supplementary Table 1.** Baseline characteristics autoantibody positive and negative patients. **Supplementary Table 2.** Frequency of initial start with methotrexate is not different for the three BMI-categories.

## Data Availability

All data relevant to the study are included in the article or uploaded as supplementary information. Additional data are available upon reasonable request.

## Abbreviations

ACPA: Anti-citrullinated peptide antibody
BMI: Body mass index
CI: Confidence interval
DAS: Disease activity score
DMARD: Disease-modifying anti-rheumatic drugs
IRR: Incidence rate ratio
MTX: Methotrexate
RA: Rheumatoid arthritis
RF: Rheumatoid factor
SJC: Swollen joint count
TJC: Tender joint count
VAS: Visual analog scale
