# Supplementary material for: Patients with obesity have more inflamed joints and higher CRP levels during the disease course in ACPA-positive RA but not in ACPA-negative RA
Source: Arthritis Res Ther. 2024 Feb 7;26:42. doi: 10.1186/s13075-023-03248-8 (PMC10848383; doi:10.1186/s13075-023-03248-8)
Supplement: Supplementary file 1 — Additional file 1: Supplementary Figure 1. Overview of patient selection. Supplementary Figure 2. Raw data of mean DAS44 and DAS-components per ACPA-subgroup per time point and per BMI-category. Supplementary Figure 3. The distribution of the patients between the different BMI-categories for ACPA-positive (A) and ACPA-negative (B) patients was similar during the 5-year follow-up. Supplementary Figure 4. Obesity associates with higher DAS and DAS-components in autoantibody positive patients and only with higher DAS44 and VAS in autoantibody negative patients. Supplementary Table 1. Baseline characteristics autoantibody positive and negative patients. Supplementary Table 2. Frequency of initial start with methotrexate is not different for the three BMI-categories. [file 13075_2023_3248_MOESM1_ESM.docx]

**Supplementary files**

[Supplementary figure 1 – Overview of patient selection 2](#_Toc153453857)

[Supplementary figure 2 – Raw data of mean DAS44 and DAS-components per ACPA-subgroup per time point and per BMI-category 3](#_Toc153453858)

[Supplementary figure 3 – The distribution of the patients between the different BMI-categories for ACPA-positive (A) and ACPA-negative (B) patients was similar during the 5-year follow-up 5](#_Toc153453859)

[Supplementary table 1 – Baseline characteristics autoantibody positive and negative patients 6](#_Toc153453860)

[Supplementary figure 4 – Obesity associates with higher DAS and DAS-components in autoantibody positive patients and only with higher DAS44 and VAS in autoantibody negative patients 7](#_Toc153453861)

[Supplementary table 2 – Frequency of initial start with methotrexate is not different for the three BMI-categories 9](#_Toc153453862)

# Supplementary figure 1 – Overview of patient selection


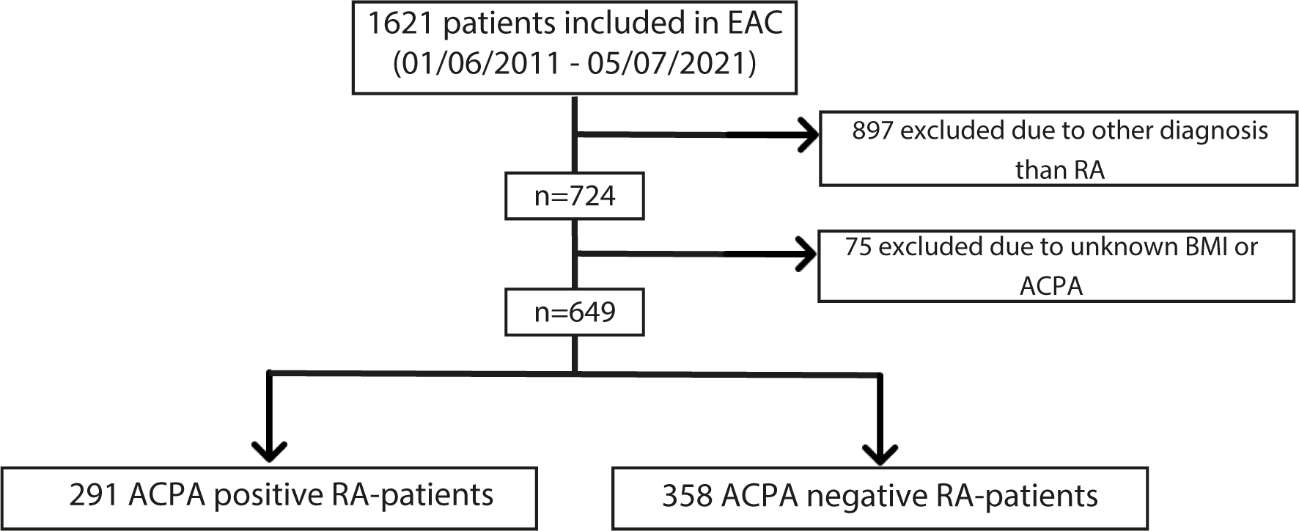


Legend: Overview of patients selected from the EAC cohort. Patients with unknown BMI or ACPA were excluded. *Abbreviations: EAC, Early Arthritis Clinic; RA, rheumatoid arthritis, BMI, body mass index; ACPA, anti-citrullinated protein antibodies.*

#
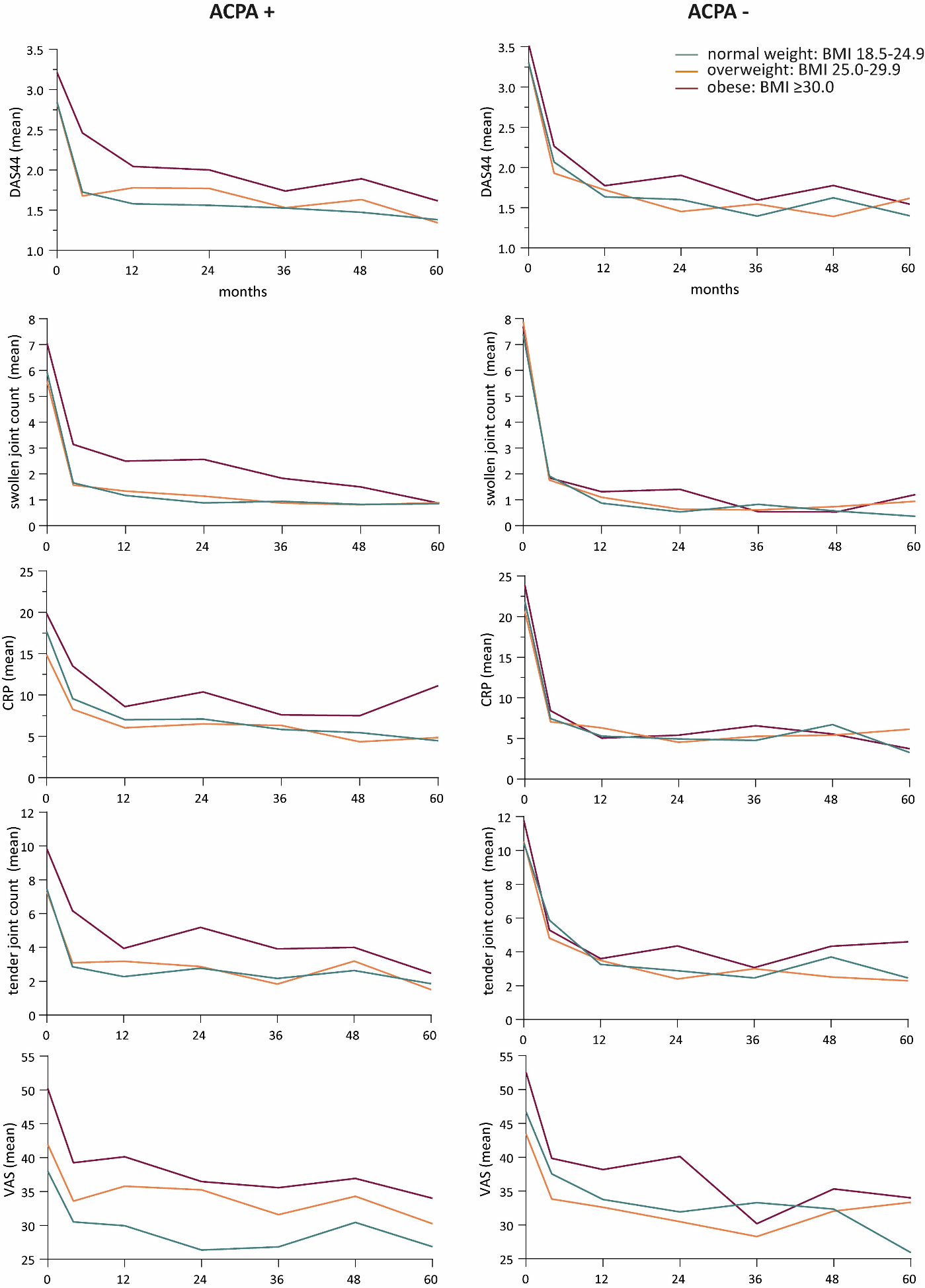
Supplementary figure 2 – Raw data of mean DAS44 and DAS-components per ACPA-subgroup per time point and per BMI-category

Legend: Trajectories of mean DAS44 and individual DAS-components during 5-years follow-up in ACPA-positive and ACPA-negative patients per BMI-category. In ACPA-positive patients both DAS44 and DAS-components are higher in obese patients compared to normal weight patients, while in the ACPA-negative RA-patients only trends towards higher DAS44, TJC and VAS can be seen in the obese patients compared to the normal weight patients. Thus the raw data show similar results compared to the results of the linear and Poisson mixed models.

# Supplementary figure 3 – The distribution of the patients between the different BMI-categories for ACPA-positive (A) and ACPA-negative (B) patients was similar during the 5-year follow-up


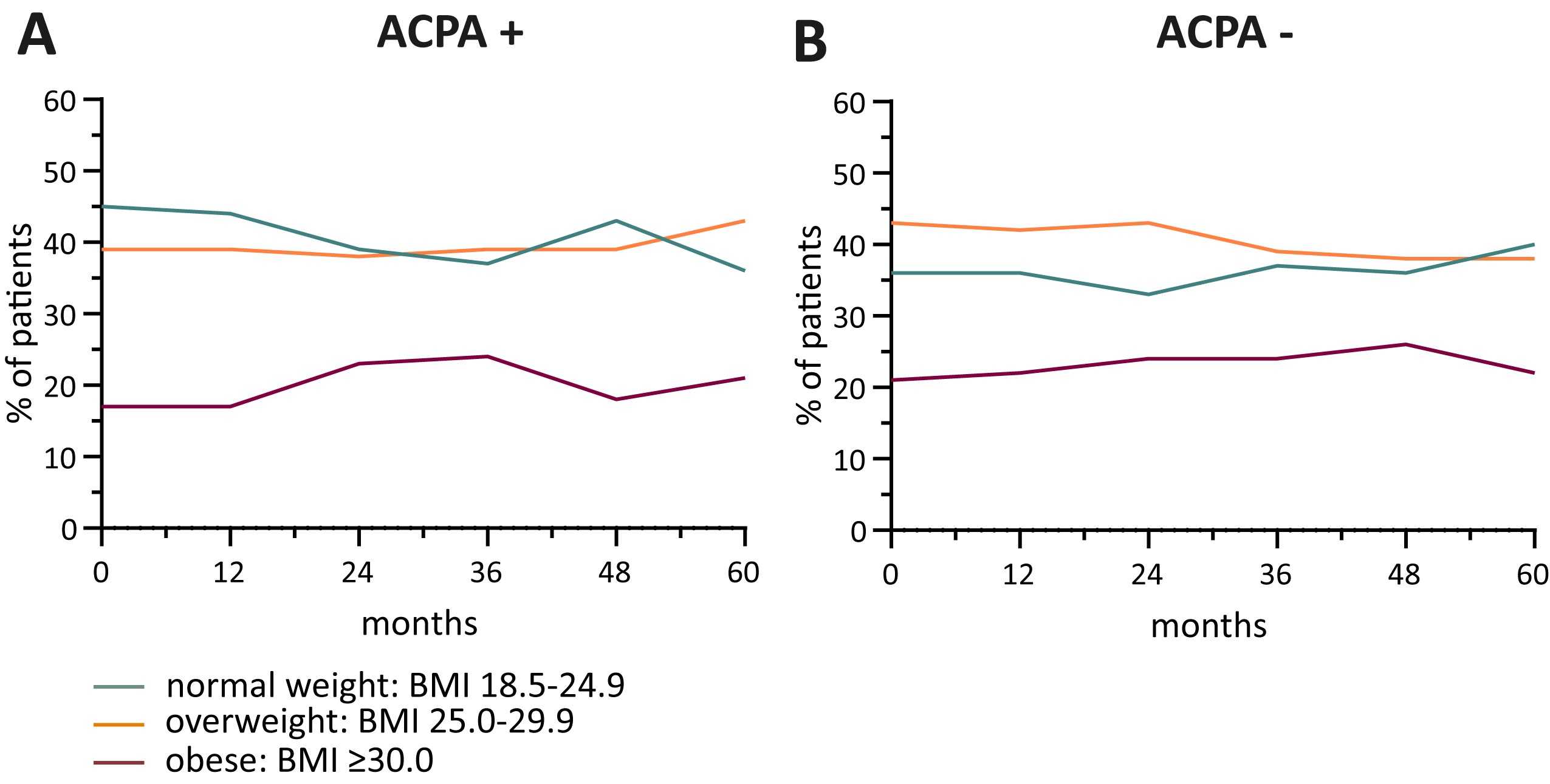


Legend: In ACPA-positive patients 284 patients were included in the analysis at baseline (A). At the subsequent follow-up duration of 1 to 5 years, data was included in analyses of respectively 238, 199, 175, 122 and 97 patients. In ACPA-negative patients 358 patients were included in the analysis at baseline (B). At the subsequent follow-up duration of 1 to 5 years, data was included in analyses of respectively 277, 197, 139, 87 and 50 patients.

As presented in the figures, the distribution of the patients between the different BMI-categories was stable during the 5-year follow-up, which was required to get unbiased results for our research questions.

# Supplementary table 1 – Baseline characteristics autoantibody positive and negative patients

|  | **Total EAC**  **N=649** | **Autoantibody pos**  **N=374** | **Autoantibody neg**  **N=259** |
| --- | --- | --- | --- |
| Age at inclusion | 59.7 (14.2) | 58.1 (14.2) | 61.4 (13.9)* |
| Female sex | 63% | 61% | 64% |
| SJC (44 joints) | 5.0 (2.0-10.0) | 5.0 (2.0-9.0) | 7.0 (3.0-11.0)* |
| TJC (53 joints) | 8.0 (4.0-13.0) | 6.0 (3.0-11.0) | 10.0 (5.0-15.0)* |
| CRP (mg/L) | 9.0 (3-24) | 8.7 (3.5-20.6) | 9.2 (3.0-25.8) |
| ACPA- and RF-positive | 37% | 64% | 0%* |
| Only ACPA-positive | 8% | 14% | 0%* |
| Only RF-positive | 13% | 22% | 0%* |
| VAS General health in mm | 40 (20-60) | 40 (20-60) | 50 (30-70)* |
| DAS44 CRP | 3.1 (2.4-3.7) | 2.9 (2.2-3.5) | 3.4 (2.7-4.0)* |
| BMI (kg/m^2^) | 26.0 (23.3-29.0) | 25.3 (22.8-28.4) | 26.6 (24.3-29.8)* |
| BMI categorized |  |  |  |
| *normal BMI 18.5-24.9* | 40% | 45% | 32%* |
| *overweight 25-29.9* | 40% | 37% | 44%* |
| *obese >29.9* | 19% | 15% | 24%* |

Legend: Data are presented as mean (SD) or median (IQR) for continuous variables, and in percentage (%) for categorical variables. n=7 patients suffered from underweight. n=16 patients were ACPA-negative and RF was unknown, therefore these patients were excluded. *significant difference between autoantibody-positive and autoantibody-negative patients (p-value<0.05). *Abbreviations:* *pos, positive; neg, negative; SJC, swollen joint count; TJC, tender joint count; CRP, c-reactive protein; VAS, visual analog scale; DAS, disease activity score; BMI, body mass index.*


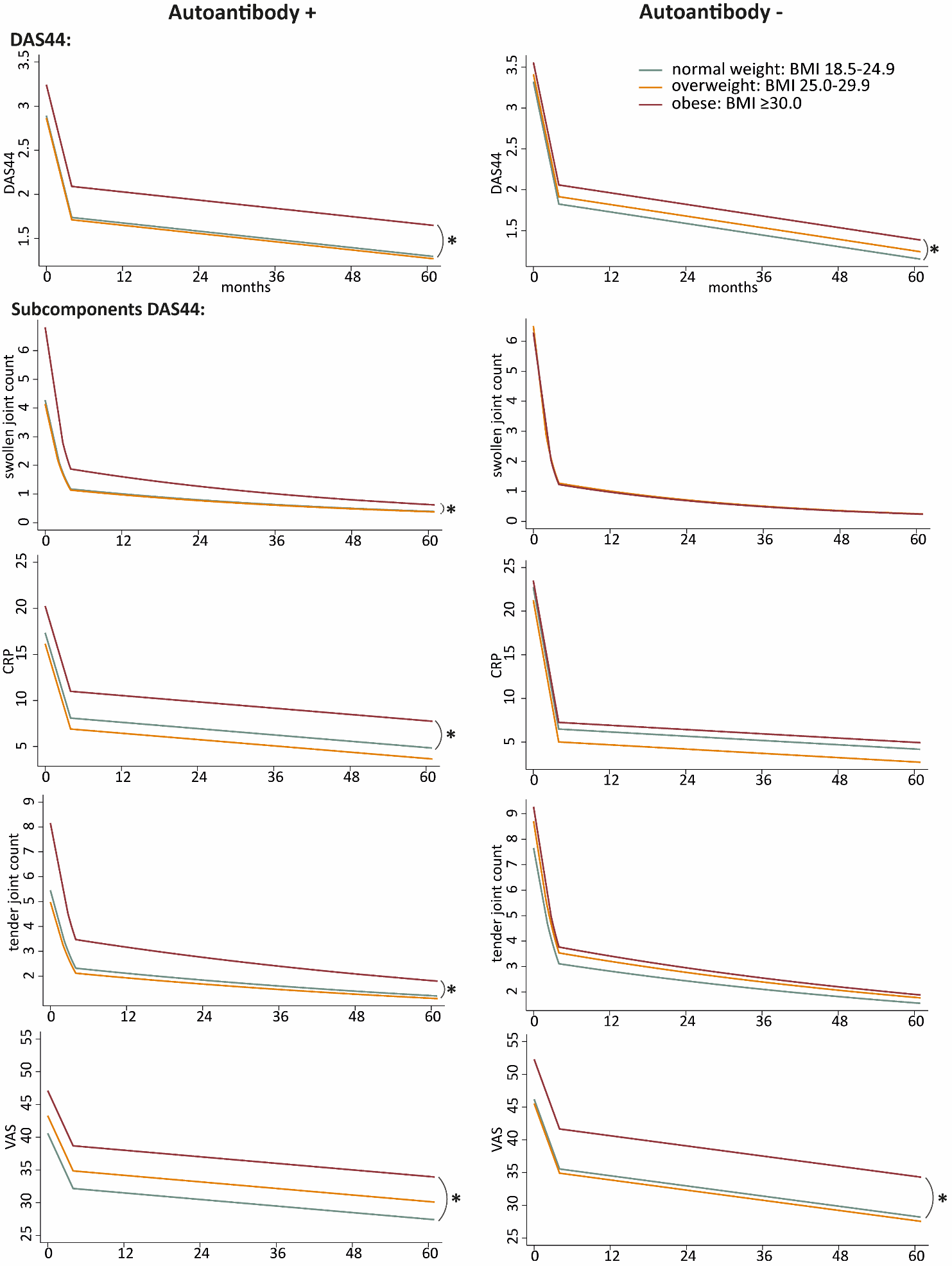
Supplementary figure 4 – Obesity associates with higher DAS and DAS-components in autoantibody positive patients and only with higher DAS44 and VAS in autoantibody negative patients**.**

Legend**:** Trajectories of DAS44 and DAS-components per BMI-category for autoantibody positive and autoantibody negative RA-patients. All trajectories are shown for mean age per autoantibody-population: 57.1 years in autoantibody positive and 60.9 years in autoantibody negative patients.

DAS: Obese autoantibody-positive patients had +0.35 units higher DAS (95%CI 0.16, 0.55), obese autoantibody-negative patients had a +0.21 (95%CI 0.01, 0.43) higher DAS compared to normal weight.

SJC: Obese autoantibody-positive patients: 59% higher SJC (IRR 1.59; 95%CI 1.22, 2.09); p-value 0.001), obese autoantibody-negative RA-patients: no significant difference (IRR0.96 95%CI 0.73, 1.24; p-value 0.78).

CRP: Obese autoantibody-positive patients: +2.9 mg/L higher CRP (95%CI 0.43, 5.43; p-value 0.02). Obese autoantibody-negative: no significant difference (β0.78 95%CI -2.51, 4.07; p-value 0.64).

TJC: Obese autoantibody-positive patients: 49% higher TJC (IRR1.49 95%CI 1.14, 1.95; p-value 0.003). Obese autoantibody-negative patients: trend towards 18% higher TJC (IRR 1.18 95%CI 0.91,1.53; p-value 0.21)

VAS: Obese autoantibody-positive patients: +6.5 units higher VAS (β6.52 95%CI 1.92, 11.1; p-value 0.005). Obese autoantibody-negative patients: +5.6 units higher VAS (β 5.60 95%CI 0.28, 10.9; p-value 0.04).

Patterns were visualized based on estimated marginal means resulting from either the linear or Poisson mixed models.

**p-value <0.05, significant difference compared to normal-weight. Abbreviations:* *BMI, body mass index. DAS, disease activity score; SJC, swollen joint count; CRP, c-reactive protein; TJC, tender joint count; VAS, visual analog scale.*

# Supplementary table 2 – Frequency of initial start with methotrexate is not different for the three BMI-categories

**Table 2A - ACPA positive RA**

|  | **Percentage of patients starting MTX within 1 year** |
| --- | --- |
| Normal weight | 91% |
| Overweight | 90% |
| Obese | 91% |

Legend: No significant differences were found in frequencies of MTX treatment between BMI-categories in ACPA-positive RA (p-value 0.98). *Abbreviations:* *BMI, body mass index. MTX, methotrexate.*

**Table 2B - ACPA negative RA**

|  | **Percentage of patients starting MTX within 1 year** |
| --- | --- |
| Normal weight | 72% |
| Overweight | 80% |
| Obese | 80% |

Legend: No significant differences were found in frequencies of MTX treatment between BMI-categories in ACPA-negative RA (p-value 0.22). *Abbreviations:* *BMI, body mass index. MTX, methotrexate.*
